# Prognostic and clinicopathological significance of FOXD1 in various cancers: a meta and bioinformation analysis

**DOI:** 10.2144/fsoa-2023-0085

**Published:** 2024-05-15

**Authors:** Xiaohan Liu, Shengyun Min, Qin Zhang, Yan Liu, Zhenhong Zou, Nanye Wang, Bin Zhou

**Affiliations:** 1 Department of general surgery, The Second Affiliated Hospital of Nanchang University, Nanchang, Jiangxi, 330006, P.R. China; 2 Nanchang University, Nanchang, Jiangxi, People's Republic of China; 3 Department of general surgery, Changzheng Hospital, Nanchang, Jiangxi, 330100, P.R. China; 4 Department of ophthalmology, The Second Affiliated Hospital of Nanchang University, Nanchang, Jiangxi, 330006, P.R. China; 5 Department of orthopedics, The Second Affiliated Hospital of Nanchang University, Nanchang, China

**Keywords:** biomarkers, cancer, *FOXD1*, meta-analysis, prognosis

## Abstract

**Aim:** To examine both predictive and clinicopathological importance underlying *FOXD1* in malignant tumors, our study adopts meta-analysis. **Methods:** We searched from PubMed, Embase, WOS, Wanfang and CNKI. Stata SE15.1 was used to calculate the risk ratio (HR) as well as relative risk (RR) with 95% of overall CIs to assess FOXD1 and overall survival rate (OS), disease-free survival rate as well as clinicopathological parameters. **Results:** 3808 individuals throughout 17 trials showed high FOXD1 expression was linked to disadvantaged OS (p < 0.001) and disease-free survival (p < 0.001) and higher TNM stage (p < 0.001). **Conclusion:** Elevated FOXD1 had worse predictions and clinicopathological parameters in most cancers. The GEPIA database findings also support our results.

## Plain language summary

*FOXD1* is a gene linked to a variety of cancers. In our article, we analyzed the results of several clinical trials in patients with different cancers. We found that when this gene is expressed in large amounts, it is often indicative of poor survival rates. From this study we can use *FOXD1* to predict the course of the disease and at the same time study its upper and lower pathways to find therapeutic drugs to treat the cancer.

## Abstract (plain language summary)

3808 individuals throughout 17 trials showed high FOXD1 expression was linked to disadvantaged overall survival (OS) (HR [hazard ratio], 1.355; 95% CI: 1.236–1.474; p < 0.001) and disease-free survival (DFS) (HR: 1.442; 95% CI: 1.030–1.854; p < 0.001) and higher TNM stage (relative risk, 1.463; 95% CI: 1.247–1.716; p < 0.001).

Regarding the association between FOXD1 expression and prognosis, increased FOXD1 expression was correlated with worse OS in adrenocortical carcinoma, colon adenocarcinoma, mesothelioma, bladder urothelial carcinoma, sarc, kidney renal papillary cell carcinoma, brain lower grade glioma, pancreatic adenocarcinoma, uveal melanoma, cervical squamous cell carcinoma and endocervical adenocarcinoma, and with worse DFS in head and neck squamous cell carcinoma, kidney renal papillary cell carcinoma, brain lower grade glioma, mesothelioma, pancreatic adenocarcinoma, uveal melanoma and kidney renal clear cell carcinoma.

FOX proteins may carry out important functions, allowing communication between biological pathways. The evolution of both vertebrates and invertebrates has been marked by the widespread presence of FOX genes, which are participating in a vast array of molecular processes as well as biological processes, containing stem cell niche maintenance, signal transduction, cell cycle regulation, metabolism control, and many others.

Studies have shown that knocking down FOXD1 could repress G3BP2 to active p53 while foster the expression of TXNIP, a downstream effector of IFN signaling leading to valid radiation treatment. On the other hand, FOXD1 is capable to bind the promoter of long non-coding RNA cytoskeleton regulator RNA (CYTOR) and SNAI2 and activates their transcription. Due to the competing of CYTOR, miR-1252 – 5p and miR-3148 are inhibited. As a consequence, lipoma preferred partner (LPP) expression regarding FOXD1-induced EMT and chemoresistance in OSCC is upregulated.

Studies confirmed FOXD1 promotes partial-EMT of LSCC cells via transcriptionally activating the expression of ZNF532.

The USA's second-leading reason for death and a significant global public health issue is cancer [1]. In recent years, numerous research has proven that many tumor markers are key for the diagnosis, therapy, and the prognosis of cancer. However, few tumor markers have been utilized in clinical research. Thus, studying dream cancer biomarkers and their effects is a promising and meaningful work.

In *Drosophila* *melanogaster*, a random mutagenesis screen was used to discover the fork head gene [2]. This research indicated that it is necessary for complete archenteron development and if lack of it would cause the foregut's homeotic transition into a head structure, giving the organism a distinctive ‘forked head’ appearance. Soon after this finding, several related genes, known as *FOX* genes, were found in a variety of species, including yeasts and humans.

The fork head box, a winged-helix DNA-binding domain of about 100 residues, is a distinctive feature of the *Drosophila* Fkh protein. While having different characteristics and activities, all Fox proteins have this particular DNA-binding domain in common. Since then, these members have been thought as fork head (or fox) proteins, owning distinctive DBD, which is identified by its homology to a region of the HNF3, is about 100 amino acids long [3]. Although being widely existed among over 100 kinds of species. The amounts of *FOX* genes change significantly between them [4,5].

*FOX* genes regulate a vast range of functions. Part of their functions involve cell cycle control [6], stem cell and stem cell niche maintenance [7,8], regulation of metabolism. The cardiac muscle, pancreas, trophectoderm, colon, kidney, lung, prostate, ovaries, brain, pancreas, thyroid, skeletal and liver, vascular tissue and immune cells all require Fox transcription factors to promote differentiation, to keep maintenance, and to carry out normal functions [9]. A Fox factor have a variety of protein domains, unique binding partners, and co-factors that can change both the specific DNA locations that are engaged and their impact on transcriptional activity [10]. Last but not least, different spatiotemporal expression patterns of multiple Fox transcription factors allow them to play a variety of roles [5]. In addition to acting as traditional transcriptional activators, Fox proteins also have pioneering roles in modulating and collaborating with other transcription factors and epigenetic effectors [11].

*FOXD1*, also called *FKHL8, FREAC-4*, belongs to conventional *FOX* gene family, being located in 5q13.2. It is important in cell reprogramming process adjustment and influences cancer cell development in many kinds of cancers. According to earlier researches, *FOXD1* could have a significant role in determining the biology of tumors. Without a question, *FOXD1* contributes significantly to tumor genesis and progression. Although many articles have revealed the correlation between *FOXD1* expression as well as prognosis, there is presently without meta-analysis assessing the diagnostic efficacy of FOXD1 for cancers. Therefore, using methodically gathered published information, our intention for this meta-analysis was to identify the connection between *FOXD1* exposure and predictive clinicopathological characteristics in various human malignancies.

## Materials & methods

### Approach for retrieving literature

We have not only cross-referenced PubMed, Embase, but also Web of Science, Wanfang and CNKI for systematic as well as exhaustive searches up to 5 May 2022. The keywords that were retrieved were as listed below: (“FOXD1” OR “Fork head box D1”) AND (“malignancy” OR “carcinoma” OR “tumor” OR “neoplasm” OR “cancer”) AND “prognosis”. In a further step, references to pertinent published literature were hand-reviewed to further pinpoint experiments that might be significant. Two researchers performed manual inspection, screening the eligible studies and extracting data separately. When there was a discrepancy, a third researcher would help to estimate, and the investigator would sentence whether differences exist. We followed the PRISMA guidelines to conduct our research.

### Choosing standards

The below listed standards were applied to ascertain which researches fulfilled the eligibility of requirements: 1) the information on the link between the expression for *FOXD1* and overall survival (OS) and disease-free survival (DFS) in participants with disease; 2) the clinico-pathological parameters were supplied; 3) the studies categorized participants into a group or groups in accordance with the expression status for FOXD1; 4) the hazard ratio (HR) and 95% CI were notified or enough other data were available to assess it. The exclusion of criteria was: 1) there were no available sufficient details to obtain the HR with 95% CI for the study; 2) expert opinion, case reports, newsletters, reviews as well as overlapping data.

### Extraction of dates & evaluation of quality

Both surveyors pulled the messages as well as the statistics from eligible for studies individually. Any dissenting comment was determined by a third surveyor. The Newcastle–Ottawa scale (NOS) to evaluate the quality of the included studies, which is suitable for evaluating case–control studies and cohort studies. The NOS system includes three sections (population selection, comparability, exposure assessment or outcome assessment) and eight items. The highest possible score is 13 stars for cohort studies and nine stars for case–control studies. NOS was adopted to appraise the methodological validity of the quality of survey that satisfied the inclusion criteria [12]. All patients with NOS score ≥6 were included in this study. The messages on tape and statistics were as below: the name of first author, publication year, tumor stage, nation, cancer category, size of sample, follow-time by months, outcome measurements, method of analysis and some other pathological parameters such as sex, age, lymph node metastasis, tumor diameter and TNM stage. In a further step, HRs and 95% CIs of OS or DFS as well as p-values were abstracted. If the Kaplan–Meier curve was only made available from the study, survival for which statistics were provided was imputed with the Engauge Digitizer version 4.1 [13,14].

### The statistical protocol

A full summary of all statistical analyses was calculated according to the results of the researches utilising Stata SE12.0 (StataCorp, College Station, TX, USA). We measured HRs and 95% CIs for OS and DFS, and relative risks (RRs) for clinicopathological parameters. Heterogeneity between individual studies was also appraised for each study as well employing the Chi-square test and the *I*^2^ statistic [15]. Because of considerable heterogeneity as ascertained by the indices of inconsistency (*I*^2^ ≥50%) and chi-square test (p ≤ 0.0), while the model of random effects was adopted to assemble researches. Bias in publications was calculated by means of Begg's funnel plot and Egger's test, where p < 0.05 was regarded as meaningful [16,17].

## Findings

### Major findings of participating research studies

**Figure 1.**
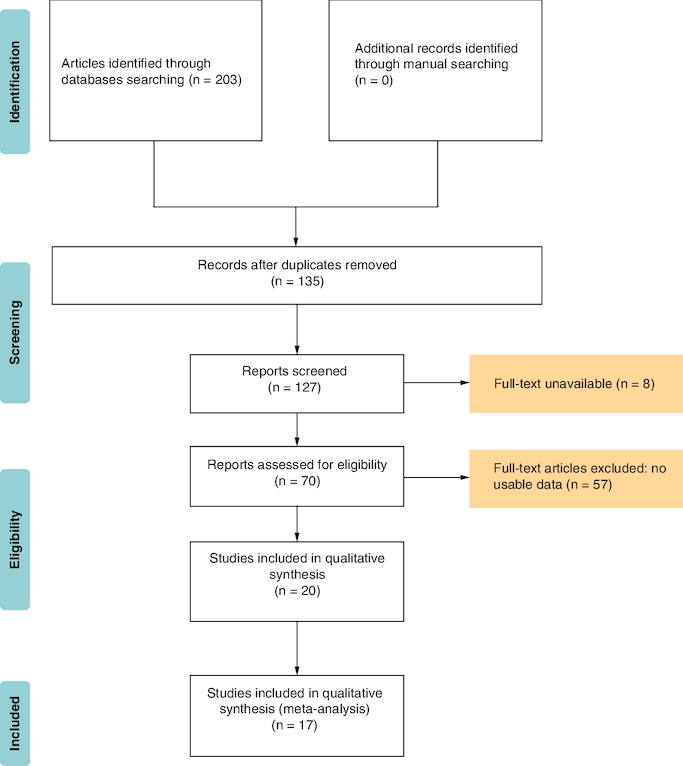
Literature retrieval and selection processes.

Following term searches through Web of Science, Embase, PubMed, CNKI and Wanfang dataset, 203 studies were in place. Then, after removing the duplication, 135 remained. By screening abstracts and data, we deleted eight articles for the full-text unavailable and these without useful data, lack a comparison and unrelated researches. The articles which got a low NOS score (<6), were removed too. Finally, there are 17 articles left, as shown in Figure 1. Seventeen studies [^18-34^] with a total of 3808 patients were found, of which one study was from Japan and 16 studies were from China. In these studies, four were used to assess the HR of DFS and all were utilized to assess the HR of OS. Table 1 contains the key details of the investigations that were recruited. In terms of sample types, the majority of studies indicated that patients had not been treated with radiation or chemical therapy.

**Table 1.** The information of enrolled articles in the meta-analysis.

Study	Year	Country	Cancer type	Sample size	Follow-up (months)	Tumor stage (I/II/III/IV)	Outcome measures	Analysis type	Sex (female/male)	n
Zhao	2014	China	BC	737	NA	NA	OS	U	737/0	5
Nakayama	2015	Japan	NCLC	90	63	68/15/6/1	OS	U/M	35/57	5
Han T	2018	China	CRC	80	60	0/7/13/20	OS	U	36/44	6
Dan Li	2018	China	NSCLC	264	NA	I–II 37 III–IV 43	OS/DFS	U/M	28/236	6
Pan F	2018	China	CRC	126	60	I–II 60 III–IV 66	OS	U	42/84	6
Jiang ZY	2018	China	GBMLGG	40	30	0/7/13/20	OS	U	18/22	6
Chang S	2019	China	CESC	292	200	NA	OS	U	NA	6
Li Z	2020	China	OSCC	58	NA	I–II 31 III–IV 27	OS	U	26/32	6
Qiu S	2020	China	HNSC	162	NA	I–II 77 III–IV 85	OS/DFS	U	37/125	6
Ding FM	2020	China	PADD	178	80	NA	OS	U	NA	5
Xie L	2020	China	CRC	72	36	14/25/25/10	OS	U	30/42	5
Zhang Q	2021	China	OSCC	146	200	NA	OS/DFS	U	NA	6
Chen Y	2021	China	OSCC	518	200	NA	OS	U	NA	6
Chen L	2021	China	EOC	93	80	I–II 19 III–IV 46	OS	U/M	93/0	6
Huang JJ	2021	China	OSCC	314	150	11/77/67/159	OS/DFS	U	96/218	6
Wang Y	2019	China	OC	120	38.5	NA	OS	U	NA	6
Wang Z	2020	China	NC	518	NA	NA	OS/RFS	U	NA	6

BC: Breast cancer; CESC: Cervical cancer; CRC: Colorectal carcinoma; DFS: Disease-free survival; EOC: Epithelial ovarian cancer; GBMLGG: Glioma; GC: Gastric cancer; HNSC: Head and neck squamous cell carcinoma; M: Multivariate; NA: Not available; NC: Nasopharyngeal carcinoma; NSCLC: Non-small cell lung carcinoma; OC: Ovarian cancer; OS: Overall survival; OSCC: Oral squamous cell carcinoma; PADD: Pancreatic adenocarcinoma; U: Univariate.

### The link between the level of *FOXD1* & OS exists

**Figure 2.**
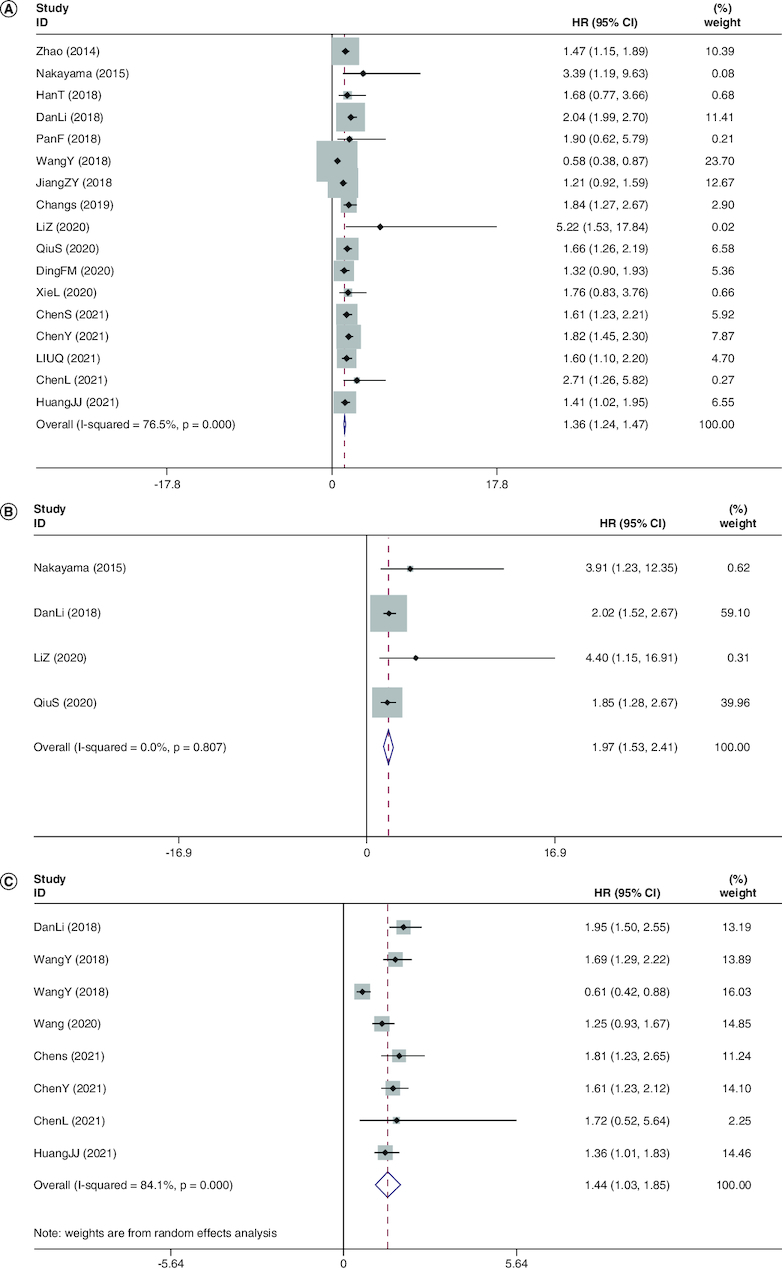
Meta-analysis of the pooled hazard ratios of overall survival and disease-free survival. **(A)** Meta-analysis of the pooled HRs of OS in cancer patients. A fixed-effect model was used for 17 studies. The pooled outcomes demonstrated a lower OS for people with most tumors with high-expression of FOXD1 if compared with those with low-expression of *FOXD1* (HR: 1.355; 95% CI: 1.236–1.474; p < 0.001). **(B)** Meta-analysis of the pooled HRs of OS in cancer patients multivariate analysis. The meta-analysis of the pooled HRs of OS in cancer patients multivariate analysis is (HR: 1.969; 95% CI: 1.530–2.408; p < 0.001). **(C)** Meta-analysis of the pooled HRs of DFS in cancer patients. A fixed-effects model was also employed for four studies. Revealing a marked connection between elevated degrees of *FOXD1* and worse DFS (HR: 1.442; 95% CI: 1.035–1.854; p < 0.001). HR: Hazard ratio; OS: Overall survival.

Among this meta-analysis, the HR for OS was validated for each of the 17 studies covering 3808 individual patients. No clear heterogeneity was apparent from one study to another (*I*^2^ = 76.5%, p < 0.001). The summarized HRs and their respective 95% CIs were derived with a fixed-effect model. The pooled outcomes demonstrated a lower OS for people with most tumors with high-expression of *FOXD1* if compared with those with low-expression of *FOXD1* (HR: 1.355; 95% CI: 1.236–1.474; p < 0.001; Figure 2A), and the meta-analysis of the pooled HRs of OS multivariate analysis is (HR: 1.969; 95% CI: 1.530–2.408; p < 0.001; Figure 2B). However, in high-grade serious human ovarian carcinoma (HGSOC), decreased performance of FOXD1 might be predictive of inferior prognosis in sufferers [33].

### The link between the level of *FOXD1* & DFS exists

There were just four surveys containing 886 individuals depicting HRs of DFS [26,29,32,35]. None of the studies noted heterogeneity between each other (*I*^2^ = 0.0%, p = 0.000). A fixed-effects model was also employed. DFS (HR: 1.442; 95% CI: 1.035–1.854; p < 0.001; Figure 2C) revealed a marked connection between elevated degrees of *FOXD1* and worse DFS.

### The link between the level of *FOXD1* & other parameters

Within this sample of 17 integrated research studies, we have strategically distributed a number of parameters. Data on the patient's clinicopathological parameters are demonstrated as pooled RRs as well as 95% CIs (Table 2). Moreover, elevated expression of FOXD1 was relevant to advanced TNM (RR: 1.463; 95% CI: 1.247–1.716; p < 0.001). Yet, no clear link was detected between *FOXD1* high expression and sex (RR: 0.965; 95% CI: 0.835–1.116; p = 0.633), age (RR: 1.002; 95% CI: 0.880–1.142; p = 0.972) or lymph node metastasis (RR: 0.966; 95% CI: 0.662–1.410; p = 0.859).

**Table 2.** Meta-analysis of the relationship between overexpressed FOXD1 and clinicopathological parameters.

	Studies (n)	Patients (n)	RR	LCI	UCI	Heterogeneity		
						*I* ^2^	*P*_h_	Model
TNM stage	8	908	1.463	1.247	1.716	5.2%	0.390	Fixed effects
Sex	7	804	0.965	0.835	1.116	52.2%	0.051	Random effects
Age	9	980	1.002	0.880	1.142	0.0%	0.925	Fixed effects
Lymph node metastasis	6	713	1.103	0.921	1.322	60.5%	0.027	Fixed effects

RR: Relative risk.

### The bias of publication

**Figure 3.**
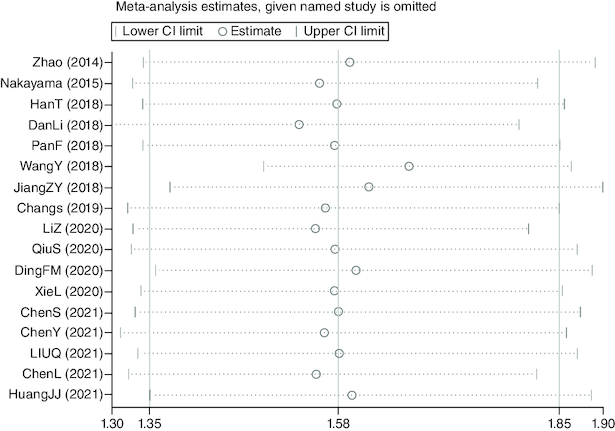
Funnel plots of publication bias about the correlation between *FOXD1* expression and hazard ratios of overall survival in the cancer patients. None publication bias for overall survival was discovered in the researches recruited by utilizing Begg's Test (Z = 0.62, *P*r > |z| = 0.538) as well as Egger's p-value (p = 0.948).

Utilizing funnel plots, the publishing bias was tested utilizing Begg's Test (Figure 3). None publication bias for OS was discovered in the researches recruited by utilizing Begg's Test (Z = 0.62, *Pr* > |z| = 0.538) as well as Egger's p-value (p = 0.948).

### Sensitivity analysis

**Figure 4.**
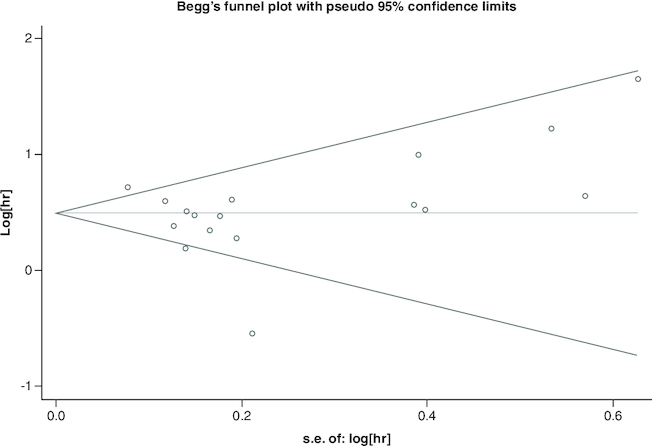
Sensitivity analysis evaluating the influence of individual study on the pooled estimate. There was no discernible impact on the pooled hazard ratio, indicating the comparative robustness of the outcome.

A sensitivity study was carried out to proceed further confirm the validity of pooled HR of OS. After excluding any particular study, the findings indicated that there was no discernible impact on the pooled HR, indicating the comparative robustness of the outcome (Figure 4).

### Validation of the results in the GEPIA database

**Figure 5.**
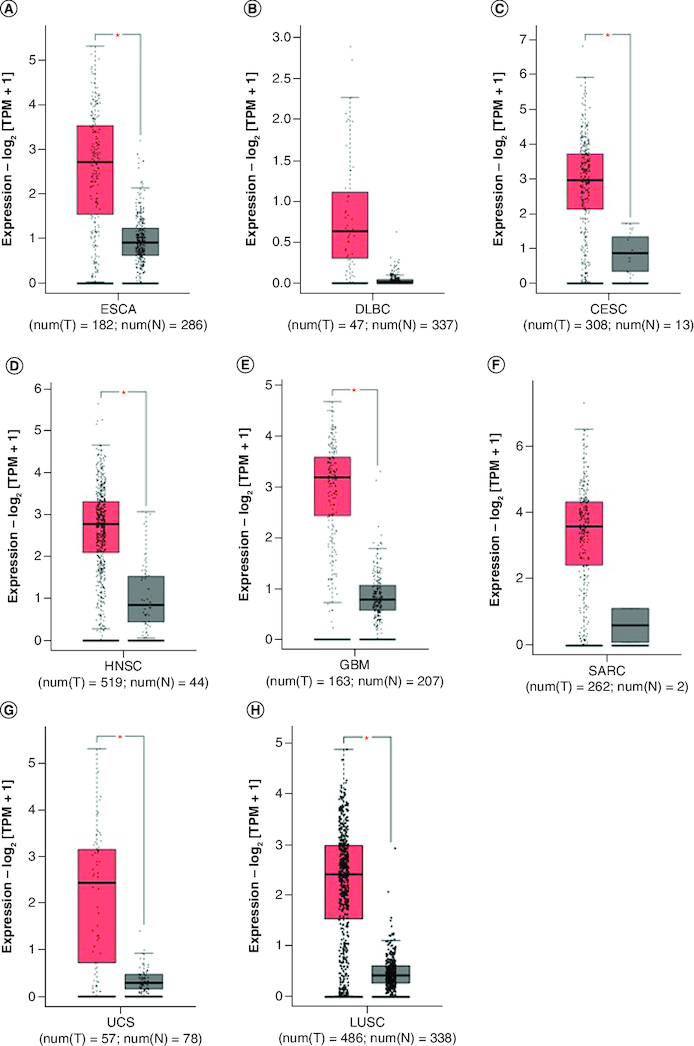
*FOXD1* expression in seven types of cancer versus normal tissue. ‘*’ |Log2Fold Change| >1 and p < 0.01. The red box plots represent FOXD1 expression in cancer tissues and the gray box plots represent FOXD1 expression in normal tissues. CESC: Cervical squamous cell carcinoma and endocervical adenocarcinoma; DBLC: Lymphoid neoplasm large B-cell lymphoma; ESCA: Esophageal cancer; HNSC: Head and neck squamous cell carcinoma; GBM: Glioblastoma multiforme; LUSC: Lung squamous cell carcinoma; SARC: Sarcoma; UCS: Uterine carcinosarcoma.

To further strengthen our conclusion, we used GEPIA on-line analysis tool to validate our results (http://gepia.cancer-pku.cn/) [36]. In terms of FOXD1 dysregulation, *FOXD1* overexpression was identified in esophageal cancer (ESCA), lymphoid neoplasm large B-cell lymphoma (DLBC), glioblastoma multiforme (GBM), head and neck squamous cell carcinoma (HNSC), lung squamous cell carcinoma (LUSC), sarcoma (SARC), cervical squamous cell carcinoma and endocervical adenocarcinoma (CESC) and uterine carcinosarcoma (UCS) (Figure 5). Regarding the association between *FOXD1* expression and prognosis, increased *FOXD1* expression was correlated with worse OS in adrenocortical carcinoma (ACC), colon adenocarcinoma (COAD), mesothelioma (MESO), bladder urothelial carcinoma (BLCA), SARC, kidney renal papillary cell carcinoma (KIRP), brain lower grade glioma (LGG), pancreatic adenocarcinoma (PAAD), uveal melanoma (UVM), cervical squamous cell carcinoma and endocervical adenocarcinoma (CESC), and with worse DFS in HNSC, KIRP, LGG, MESO, PAAD, UVM and kidney renal clear cell carcinoma (KIRC) (Figure 6A & B). These results support our results and indicate that *FOXD1* could be a novel prognostic biomarker for various cancers.

Figure 6.Validation of the prognostic effect of FOXD1 on cancer patient overall survival and disease-free survival based on the GEPIA online database.**(OS section)** Validation of the prognostic effect of FOXD1 on cancer patient overall survival (OS) based on the GEPIA online database. **(A)** OS plot of FOXD1 in adrenocortical carcinoma. **(B)** OS plot of FOXD1 in bladder urothelial carcinoma. **(C)** OS plot of FOXD1 in cervical squamous cell carcinoma and endocervical adenocarcinoma. **(D)** OS plot of FOXD1 in colon adenocarcinoma. **(E)** OS plot of FOXD1 in kidney renal papillary cell carcinoma. **(F)** OS plot of FOXD1 in brain lower grade glioma. **(G)** OS plot of FOXD1 in mesothelioma. **(H)** OS plot of FOXD1 in pancreatic adenocarcinoma. **(I)** OS plot of FOXD1 in sarcoma. **(J)** OS plot of FOXD1 in uveal melanoma (DFS section). Validation of the prognostic effect of FOXD1 on cancer patient disease-free survival (DFS) based on the GEPIA online database. **(A)** DFS plot of FOXD1 in head and neck squamous cell carcinoma. **(B)** DFS plot of FOXD1 in kidney renal clear cell carcinoma. **(C)** DFS plot of FOXD1 in kidney renal papillary cell carcinoma. **(D)** DFS plot of FOXD1 in brain lower grade glioma. **(E)** DFS plot of FOXD1 in mesothelioma. **(F)** DFS plot of FOXD1 in pancreatic adenocarcinoma. **(G)** DFS plot of FOXD1 in uveal melanoma.

**Figure:**
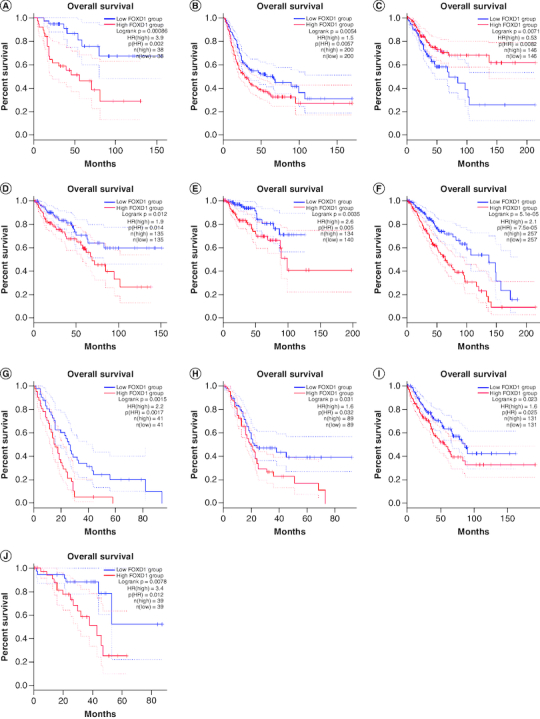

**Figure:**
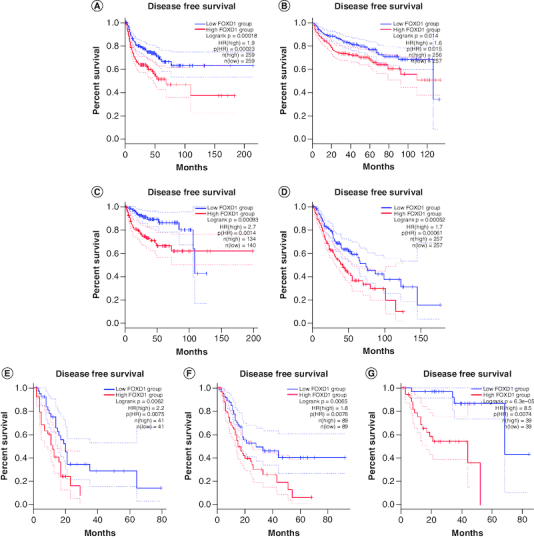

## Discussion

For the first time, *FOXD1* was found and described in the forebrain neuroepithelium [37], where it was connected to several important the development of the kidney and vitreous retina and embryo insertion. It is expressed in a diverse kind of tissues and the cellular compartment, including the kidney, the testis, the pituitary gland, the central nervous system, the mesenchyme of the facial growing centers, the neuroepithelial cell of the anterior thalamus and hypothalamus, cavernous muscle cells, peri-pulmonary cells as well as the placenta, and is overexpressed in some tumors [38].

FOX proteins are a class of evolutionarily conserved transcriptional factors that regulate numerous cellular pathways throughout the evolution of cancer, including the mitogen-activated protein kinase pathway, Wnt pathway, TGF-pathway [39]. There is growing evidence that in cellular networks, these FOX proteins may carry out important functions, allowing communication between biological pathways. The evolution of both vertebrates and invertebrates has been marked by the widespread presence of *FOX* genes, which are participating in a vast array of molecular processes as well as biological processes, containing stem cell niche maintenance, signal transduction, cell cycle regulation, metabolism control and many others.

In head and neck squamous cancer, elevated expression of *FOXD1* leads to more terrible immunologic function [40]. *FOXD1* could regulate EGFR expression to modulate the effect of cetuximab in HNSC, thus making the prognosis worse [41]. The two main types of treatment for oral epithelial cell carcinoma are chemotherapy and radiation. * FOXD1* has some negative effects on both therapies. Studies have shown that knocking down *FOXD1* could repress G3BP2 to active p53 while foster the expression of TXNIP, a downstream effector of IFN signaling leading to valid radiation treatment. On the other hand, *FOXD1* is capable to bind the promoter of long non-coding RNA cytoskeleton regulator RNA (CYTOR) and SNAI2 and activates their transcription. Due to the competing of CYTOR, miR-1252–5p and miR-3148 are inhibited. As a consequence, lipoma preferred partner (LPP) expression regarding FOXD1-induced EMT and chemoresistance in OSCC is upregulated [29,30,42]. Studies confirmed *FOXD1* promotes partial-EMT of LSCC cells via transcriptionally activating the expression of ZNF532 [43]. By regulating histone H3 *FOXD1* controls the cell cycle in ccRCC cells of colorectal cancer (CRC). In addition, using both *FOXD1* and *Plk2* may as a novel biomarker can better predict poor prognosis in colorectal cancer. Patients who have high expression of both FOXD1 and Plk2 tend to face the most terrible survival outcomes [44]. In non-small cell lung cancer, FOXD1 translocation to the nucleus to activate Gal-3 expression is inhibited by inactivation of ERK or depletion of ERK1 or ERK2 [45]. *FOXD1* downregulation decreased the immune protective function since resting M1 macrophages, memory CD4^+^ cell, monocytes were considerably lower in the high *FOXD1* expression group than in the low *FOXD1* expression group [46].

In breast cancer (BC), the FOXD1-dependent RalA-ANXA2-Src complex promotes CTC formation in breast cancer. The FOXD1-RalA-ERK1/2 signaling cascade mediates CTC formation and BC cell migration. *FOXD1* also enhances BC proliferation and chemoresistance [47]. In gliomas through controlling aerobic glycolysis, *FOXD1* subsequently increases GLUT1 expression and finally promotes PC cell proliferation, metastasis, invasion, and cell proliferation [48].

In our work, we observed that *FOXD1*‘s advanced translation was linked to OS (HR: 1.355; 95% CI: 1.236–1.474; p < 0.001) and poorer DFS (HR: 1.969; 95% CI: 1.53–2.408; p < 0.001). High *FOXD1* expression in most cancers indicates a bad prognosis and shorter survival time. However, in highly differentiated serous ovarian cancer, the opposite is true. Longer survival times and better prognoses were associated with greater *FOXD1* expression. In highly differentiated serous ovarian cancer, the study discovered a mechanism that miR-30a-5p and miR-200a-5p targeted *FOXD1*. *FOXD1* suppressed the growth of ovarian cancer cells and slowed the progression of ovarian cancer by boosting p21 expression in a p53 independent manner [33]. Why the relationship between *FOXD1* expression levels and prognosis in HGSOC is different from other tumors? Whether there are predictive relationships in other tumor subtypes that differ from the predominant type may be a potential direction for future research.

There is also a correlation between *FOXD1* and pathological parameters of some cancers. For example, higher *FOXD1* expression was obviously related with lymph node metastasis, tumor size and advanced stage, in colorectal cancers and nasopharyngeal [49], non-small cell lung cancer [19]. In HNSCC, overexpression of *FOXD1* significantly associated with clinical stage and lymph node metastasis. In our study, high level of expression of *FOXD1* was linked to advanced TNM (RR: 1.463; 95% CI: 1.247–1.716; p < 0.001). Yet, none clear link was detected between *FOXD1* overexpression as well as sex (RR: 0.965; 95% CI: 0.835–1.116; p = 0.633), age (RR: 1.002; 95% CI: 0.880–1.142; p = 0.972) or lymph node metastasis (RR: 0.966; 95% CI: 0.662–1.410; p = 0.859).

Regarding the association between *FOXD1* expression and prognosis, increased *FOXD1* expression was correlated with worse OS in adrenocortical carcinoma (ACC), colon adenocarcinoma (COAD), mesothelioma (MESO), bladder urothelial carcinoma (BLCA), SARC, kidney renal papillary cell carcinoma (KIRP), brain lower grade glioma (LGG), pancreatic adenocarcinoma (PAAD), uveal melanoma (UVM), cervical squamous cell carcinoma and endocervical adenocarcinoma (CESC), and with worse DFS in HNSC, KIRP, LGG, MESO, PAAD, UVM and KIRC.

This meta-analysis has a pile of shortcomings that should be considered. Because of a certain amount of the factors, we were unable to provide additional data to confirm the carcinoma connection seen, containing the fact that only 17 studies totaling 3808 individuals were recruited for this meta-analysis. And even more rarely were studies evaluated in the DFS and clinicopathological parameters subgroups of analysis. Second, it's important to exercise caution when using many survival statistics built on the Engauge Digitizer. Third, one of the main drivers of variability may be because the cut-off value for the definition of the *FOXD1* high-level expression is not a fixed value. Fourth, several retrospective research are included in this meta-analysis.

## Conclusion

Individuals with elevated *FOXD1* expression had poorer survival predictions and worse clinicopathological parameters in most of cancers compared with patients with low *FOXD1* expression. However, may indicate a poor prognosis in patients with high-grade, severe human ovarian cancer.
